# The Antimicrobial Potential of Bacteria Isolated from Honey Samples Produced in the Apiaries Located in Pomeranian Voivodeship in Northern Poland

**DOI:** 10.3390/ijerph15092002

**Published:** 2018-09-14

**Authors:** Magdalena Pajor, Randy W. Worobo, Sławomir Milewski, Piotr Szweda

**Affiliations:** 1 Department of Pharmaceutical Technology and Biochemistry, Faculty of Chemistry, Gdańsk University of Technology, ul. G. Narutowicza 11/12, 80-233 Gdańsk, Poland; magdalena.pajor@pg.edu.pl (M.P.); slawomir.milewski@pg.edu.pl (S.M.); 2 Department of Food Science, Cornell University, Ithaca, NY 14853, USA; rww8@cornell.edu

**Keywords:** honey, antimicrobial activity, growth inhibition potential, *Bacillus*

## Abstract

The principal objective of this study was to determine whether the honeys produced in apiaries located in Pomeranian Voivodeship (Northern Poland) contain bacteria producing metabolites with growth inhibition potential against important human and animal pathogens. The pathogens included *Staphylococcus aurues*, *Staphyloccocus epidermidis*, *Escherichia coli*, *Listeria monocytogenes*, *Pseudomonas aeruginosa,* and *Candida albicans*. From 12 samples of honey, 163 strains of bacteria were isolated. Activity against reference staphylococci: *S. aurues* ATCC 25923; *S. aureus* ATCC 29213; *S. epidermidis* 12228 was observed in 33 (20.3%), 38 (23.3%), and 41 (25.1%) isolates, respectively. High inhibitory activity was also found against *Listeria monocytogenes* ATCC 7644 in 34 strains (20.9%). Activity against *Candida albicans* ATCC 10231 and especially Gram-negative bacteria: *Pseudomonas aeruginosa* ATCC 27857 and *Escherichia coli* ATCC 25922 was rarely observed. Production of metabolites exhibiting activity against the three pathogens mentioned above was confirmed for 13 (7.8%), 3 (1.8%), and 2 (1.2%) isolates, respectively. Forty-six isolates were selected for further analysis. Within this group, metabolites synthesized by 18 producing strains (39.13%) inhibited growth of only one of the reference strains of pathogenic microorganisms. However, 14 (30.44%), 8 (17.39%), and 6 (13.04%) strains produced agents active against three, two, and four pathogens, respectively. Sequencing of the 16S rRNA gene revealed that 80.4% of these 46 producing strains belong to the genus Bacillus. However, some producing strains belonging to the genus of *Peanibacillus*, *Lysinibacillus*, *Microbacterium,* and *Staphylococcus* were also identified. Furthermore, the analysis of the sequences of 16S rRNA, as well as RAPD-PCR, exhibited a significant diversity in the strains tested, even in the case of bacteria isolated from the same honey (and classified to the same genus, usually *Bacillus* spp.). This observation suggests environmental origin (nectar, water, or pollen) of the producing strains. The research carried out confirmed that honey produced in Northern Poland is a promising source of strains of bacteria producing metabolites with antimicrobial activity.

## 1. Introduction

With respect to health benefits, honey is definitely among the most valuable natural products. For centuries it has been used not only as a sweet, tasty, and popular food product but also as one of the most important agents of so-called traditional medicine. The research from the last 50–70 years provides clear confirmation of the positive effects of honey on human health [1]. The antibacterial, anti-inflammatory, apoptotic, and antioxidant properties of honey are discussed in the management of disease conditions in various medical applications. Recent studies report a strong interest in the application of honey as a component of effective biologic wound dressings with multiple bioactivities—especially focusing on its high antibacterial potential [2]. Recently, it has been proved that the enzymatic generation of hydrogen peroxide and the presence of some phytochemicals (mostly polyphenols) are crucial for the antimicrobial potential of honey. However, we do not yet have a complete understanding of the mechanism of the antimicrobial properties of honey. The issue has been examined in more detail in other publications [3,4,5,6,7]. High osmotic pressure (a consequence of the high sugar content—about 80% of the mass of the product), and the low pH of the product additionally support its antimicrobial potential and create a hostile environment for the majority of microflora. However, the investigations of many authors confirm that honey is not a sterile product. The microorganisms that have been identified in samples of honey produced in various geographical areas include both pathogenic and beneficial species. Microorganisms capable of withstanding the honey’s intrinsic harsh growing conditions are derived from primary or secondary sources of microbial contamination [8]. Plant-associated microorganisms residing on flower surfaces, in nectars, pollen, soil, and water are the most predominant bacteria in honey. Moreover, the digestive tract of honeybees has been found to be an important source of microbial contamination of honey. During the process of honey production, bees introduce some bacteria from their gut microbiota into the nectar. There is a broad range of bacteria transferred from the digestive tract to the product, including: *Lactobacillus rigidus*, *Bacillus* spp., *Streptococcus* spp., *Clostridium* spp., and Gram-negative bacteria. Furthermore, *Achromobacter* spp., *Citrobacter* spp., *Enterobacter* spp., *Flavobacterium* spp., *Klebsiella* spp., *Proteus* spp., *Pseudomonas* spp. have also been identified [9,10]. However, it should be noted that the composition of bees’ gut microbiota depends on many factors, e.g., during the flowering season of rape Wang et al. [11] indicated the *Bacillus* group as dominant bacteria in honey bee stomachs. Recent studies on bacteria associated with gut microflora have revealed that the gastrointestinal tract of honey bees is a favorable environment not only for the growth of Lactic Acid Bacteria (LAB) like *Lactobacillus* spp. and *Enterococcus* spp., but also for Fructophilic Lactic Acid Bacteria (FLAB) [12], predominantly *Lactobacillus kunkeei* [13]. The main difference between LAB and FLAB is the sugar preferred by the bacteria from each group as a growth substrate—glucose or fructose. The digestive tract of Slovakian honey-bees was found by Kačániová et al. [14] to be mainly populated by anaerobic, rather than aerobic bacteria: *Coliforms*, *Enterococci*, *Staphylococci*, *Bacillus* spp., *Pseudomonas* spp., microscopic fungi and yeast. Additionally, the microflora of the gastrointestinal tract of summer and winter bees has been shown to diversify, thus, a broad spectrum of different microorganisms can be transferred from bees’ gut to honey.

Microorganisms coming from post-harvest sources, including human, equipment, and even dust, are considered as second source contamination and they can be divided into three categories [15]: spore-forming microorganisms commonly found in honey; microorganisms generally used as indicators of hygienic quality; and microorganisms whose presence might infer specific conditions, such as germination [16]. In this regard, the community of microorganisms residing in honey is a combination of bacteria, yeast, and mold which may vary under certain conditions.

In our previous studies, we confirmed the high antibacterial (especially antistaphylococcal) potential of honeys produced in Polish apiaries [4,17]. In the present study, we investigated if these products contain bacteria producing antimicrobial agents. The results obtained confirm the observations of authors from other laboratories suggesting that honey should be considered as a reservoir of bacteria which have food preservative or even chemotherapeutic properties [18,19,20,21,22].

## 2. Materials and Methods

### 2.1. Honey Samples and Isolation of Bacterial Strains

The samples of multiflower (*n* = 9), buckwheat (*n* = 2), and honeydew (*n* = 1) honeys were provided by beekeepers from Pomeranian Voivodeship, in Northern Poland and were stored in dark conditions at room temperature with no signs of alteration. Honeys were diluted with sterile distilled water in a 1:1 (*v/v*) ratio and 1 mL of each suspension was streaked on a Petri dish of twenty centimeters diameter containing a solid growth medium (Luria-Bertani—LB agar). Petri dishes were then incubated over night at 37 °C. The growing colonies were counted and the level of microbial contamination (CFU/mL) of the honey samples was calculated. Thereafter, the individual colonies were transferred by sterile pipette tip onto fresh Petri dishes with the same solid growth medium (LB agar) and incubated over night at 37 °C. In this manner, a collection of bacteria isolated from honey samples was obtained for further researches. In the case of products exhibiting higher level of microbial contamination (CFU/mL ≥ 2 × 10^2^), 20 randomly selected colonies with a different morphological appearance were selected for further research.

### 2.2. Growth Inhibitory Assay

**Figure 1:**
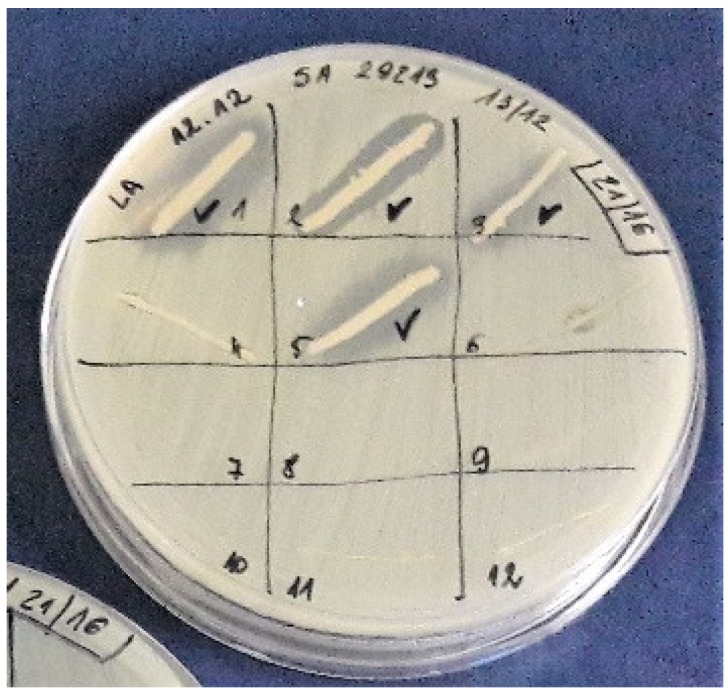
The growth inhibition zones of *Staphylococcus aureus* ATCC 25923.

Isolated colonies from the collection were transferred by sterile pipette tip (in the form of a spot or short line—0.5 to 1 cm) onto Petri dishes with solid growth medium (LB agar) inoculated by reference strains: *Staphylococcus aurues* ATCC 25923; *S. aureus* ATCC 29213; *S. epidermidis* 12228; *Escherichia coli* ATCC 25922; *Listeria monocytogenes* ATCC 7644; *Pseudomonas aeruginosa* ATCC 27857; and *Candida albicans* ATCC 10231. Inoculation was performed by streaking with a sterile cotton swab soaked in a suspension of each tested reference strain (final optical density of each suspension OD_600_ = 0.1). Plates were incubated over night at 37 °C. The observed halo zones (Figure 1) indicated the growth inhibition of reference strain and predestined colonies of bacteria isolated from honey for further research.

### 2.3. The Identification of Bacterial Species Producing Antibacterial Metabolites

The identification of the producing strains (isolates recognized as producers of antimicrobial metabolites) was carried out by sequencing of the 16S rRNA gene. The DNA was isolated using Genomic Mini AX (A&A Biotechnology, Aleja Zwycięstwa 96/98, 81-451 Gdynia) according to the protocol purchased from the manufacturer of the kit. PCR (Polymerase Chain Reaction) amplification of the target gene was determined with a pair of primers:

rP1 5’ CCCGGGATCCAAGCTTAGAGTTTGATCCTGGCTCAG 3’
fD2 5’ CCGAATTCGTCGACAACACGGCTACCTTGTTACGACTT 3’

and the methodology described by the group of Weisburg [23]. Sequencing of the amplified product was carried out by Macrogen (Meibergdreef 31, 1105 AZ Amsterdam the Netherlands). The amplified gene coding for 16S rRNA was purified using the enzymatic Post-PCR Immediate Cleanup (EPPiC) purification kit (A&A Biotechnology, Aleja Zwycięstwa 96/98, 81-451 Gdynia) following the protocol provided by the producer.

### 2.4. DNA Sequence Analysis

The sequence analyses were performed with BLAST (Basic Local Alignment Search Tool). The phylogenetic tree was constructed from the 16S rRNA sequences in Phylogenetic Tree Builder Tool in NCBI (National Centre for Biotechnology Information) Genome Workbench from previously generated FASTA sequences in Snap Gene 4.1.9. The phylogenetic tree was constructed using neighbor-joining method and sorted by distance. Multiple Sequence Comparison by Log-Expectation (MUSCLE v 3.8.31) for medium alignments was also performed using scoring method by log-expectation.

### 2.5. Genetic Differentiation of Producing Strains

Because of the high similarity or even identity of the sequences of genes coding for 16S rRNA, the genetic differentiation of producing strains was additionally investigated with the RAPD-PCR (Random Amplification of Polymorphic DNA) method. Each PCR reaction was performed in the final volume of 50 μL. The reaction mixture was prepared according to the protocol provided by the producer of the ready to use PCR MIX (A&A Biotechnology, Aleja Zwycięstwa 96/98, 81-451 Gdynia, Poland) with primers AB: 5’(GACA)_4_ (Sigma Aldrich, St. Louis, MI, USA). The amplification reaction was carried out in Mastercycler (Eppendorf, Hamburg, Germany) and the conditions were: initial denaturation at 95 °C for 3 min following by 34 cycles, denaturation at 95 °C for 30 s, annealing at 42 °C for 30 s, elongation at 72 °C for 30 s, finally the reaction mixture was incubated at 72 °C for 300 s and cooled to room temperature.

Electrophoretic separation of amplified PCR fragments was performed in 2% agarose gel (Sigma Aldrich) at a voltage of 130 V for 30 min.

## 3. Results

### 3.1. Bacterial Content in Honeys and Isolation of Producing Strains

The investigated samples of honey presented different levels of microbial contamination (only aerobic or facultative aerobic bacteria were taken into account).

In the cases of nine products (75%), the total number of bacteria was below 10^2^ CFU/mL, in one, it was 3.4 × 10^2^ CFU/mL, and the two last honeys contained 3.04 and 6.27 × 10^3^ of bacterial cells in a volume of 1 mL (Table 1).

All the products investigated contained at least one isolate exhibiting activity against one of the reference strains (Table 1).

One hundred and sixty-three isolates were screened for production of antimicrobial compounds—all strains recovered from honeys with a level of contamination lower than 10^2^ CFU/mL (nine samples) and 20 randomly selected isolates of each of the products containing more than 10^2^ CFU/mL (three honeys). The results concerning production of metabolites inhibiting the growth of *staphylococci* were particularly promising: 33 strains inhibited the growth of *S. aureus* ATCC 25923, 38 inhibited the growth of *S*. *aureus* ATCC 29213, and 41 isolates affected the growth of *S*. *epidermidis* ATCC 12228. Overall, from 10% to 40% of isolates from seven products investigated in this research exhibited activity against these pathogenic microorganisms. Only two honeys (24/16 and J.K/2018) did not contain any bacteria producing antistaphylococcal agents.

The isolates obtained in the present study also exhibited a high activity against *L. monocytogenes* ATCC 7644 (38 active strains). Importantly lower activity was observed against Gram-negative bacteria: *E. coli* ATCC 25922 (two active strains) and *P. aeruginosa* ATCC 27857 (three active strains). Lower susceptibility was also observed in the case of *C. albicans* with 13 isolates effectively inhibiting the growth of these pathogenic yeasts (Table 1).

### 3.2. Species Identification and Genetic Differentiation of “Producing” Strains

The most outstanding strains (strains that exhibited activity against at least one of the reference bacteria) out of a total number of 46 were selected for further species identification. The 16S rRNA gene sequences were analyzed using BLAST software and revealed that 37 out of 46 isolated strains (80.4%) belong to the genus *Bacillus*. The sequences of the 16S rRNA gene of different species of *Bacillus* spp. are characterized by a high level of similarity or even identity. Thus, in many cases it was not possible to classify the isolate to a particular species. As a consequence, in Table 2, several isolates do not have a final species classification—two or more of the most possible species are proposed. In general *Bacillus* spp. isolates have been classified into six different species: *B. pumilus; B. licheniformis; B. safensis; B. zhangzhouensis; B. altitudinis; B. xiamenensis.* The growth inhibition of Gram-positive reference strains is mainly observed in the case of *Bacillus* spp. Other than them, producing strains were also recognized as *Peanibacillus* spp.; *Lysinibacillus* spp.; *Microbacterium* spp.; and *Staphylococcus* spp.

**Figure 2:**
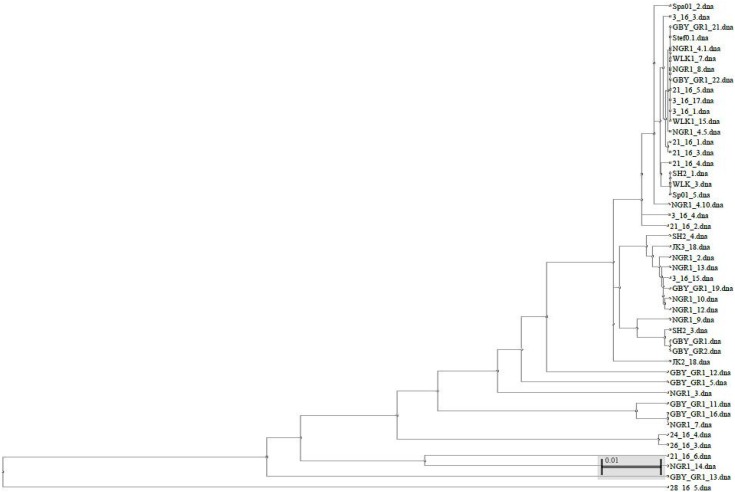
Phylogenetic tree of 46 microorganisms isolated from Polish honeys based on 16S rRNA gene sequences.

The phylogenetic tree constructed from the 16S rRNA sequences is shown in Figure 2. Furthermore, the detailed analysis of the 16S rRNA sequences of the tested producing strains exhibited a significant diversity even in the case of bacteria isolated from the same honey (and classified to the same genus, usually *Bacillus* spp.). In this regard, the environmental origin (nectar, water, pollen) of the producing strains is evident (Figure 2).

**Figure 3:**
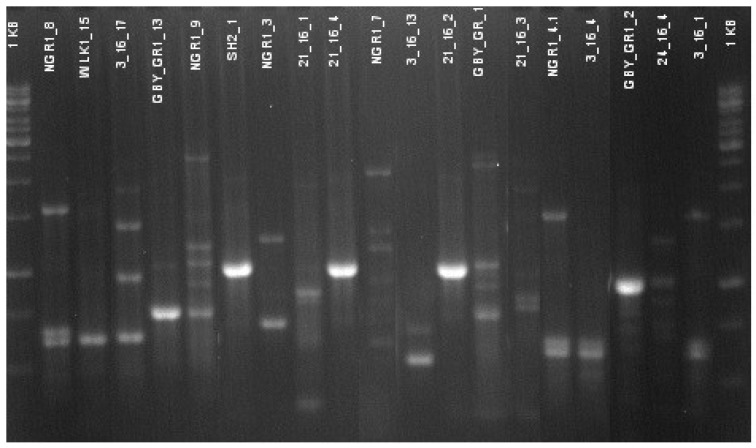
Electrophoretic profiles of amplified DNA fragments for several isolates selected.

High genetic diversity within the “producing” strain population has also been confirmed with the RAPD-PCR method. An example of differences in electrophoretic profiles of amplified DNA fragments for several isolates selected is shown in Figure 3.

## 4. Discussion

Different authors confirm that the level of contamination of honey with aerobic bacteria is relatively low. It can be explained by the fact that high osmotic pressure, low pH, and the presence of many agents, including hydrogen peroxide, bee defensin-1 (an antibacterial peptide belonging to the insect defensin group, bees use this peptide for antimicrobial protection of honey and royal jelly [24]), and phytochemicals, effectively inhibit the growth and reproduction of bacteria in honey [5]. In most reports presented to date, the total number of living cells of aerobic bacteria per one gram of honeys varies between zero to tens of thousands [25,26,27,28]. The results of the present study are in agreement with these observations. Only in the case of three out of twelve products (25%) was the level of contamination of the honey higher than 10^2^ CFU/mL. The aforementioned microflora of honey include both pathogenic and probiotic microorganisms. The main purpose of our study was the selection of bacteria producing antimicrobial agents. Successful selection of potential producers of antimicrobials from honey has been reported by several authors from different geographical locations. Lee et al. [29] screened six US honeys and two manuka honeys originating from New Zealand. The researchers reported that 92.5% of a total of 2398 strains exhibited antimicrobial activity against at least one of the tested microorganisms: *Bacillus subtilis* ATCC 6633, *Bacillus cereus* F4552, *L. monocytogenes* F2-586 1053, *S. aureus* ATCC 9144, *E. coli* O157:H7 ATCC 43889, *Salmonella enterica, Serovar Enteritidis* and *Vibrio parahaemolyticus* G1-166 (O3: K6). In another study, Lee and Lee [30] isolated 327 strains of bacteria from seven Korean honeys and found 109 of them (33.3%) as active against five foodborne pathogens. Wahab and et al. [31] isolated three strains of *Bacillus* spp. from African honeys (one from Nigeria and two from Egypt) that effectively produced metabolites with an antibacterial activity against 13 indicator microorganisms, including important human and animals pathogens: *S. aureus, E. coli, Salmonella typhimurium,* *P. aeruginosa,* *C. albicans* and *Aspergillus niger.* The group of Aween [19] reported that honey from Malaysia, Libya, and Saudi Arabia contained strains of *Lactobacillus acidophilus* producing compounds with antibacterial activity against Gram-negative bacteria: *S. typhimurium*, *E. coli,* and *Enterobacter aerogenes.* Four *Enterococcus faecium* strains secreting antimicrobial agents active against *L. monocytogenes* have been isolated from honeycombs in Argentina by the group of Ibarguren [32].

The results of the present research have demonstrated that honey of different floral origins produced in apiaries located in Northern Poland is a potent source of bacteria capable of synthesizing antimicrobial substances. Gram-positive bacteria were found to be less resistant to the antibacterial compounds than Gram-negative. Furthermore, the diversity of *Bacillus* spp. (confirmed with RAPD-PCR and analysis of sequences of genes coding for 16S rRNA genes) indicates their environmental background. Similar results have been shown in several studies.

Among 433 honey samples collected in Argentina in different years by López et al. [33], 114 (27%) yielded *B*. *cereus*, 52 (12%) yielded *B*. *megaterium*, 5 (1%) yielded *Bacillus mycoides,* and 3 (0.7%) yielded *Bacillus thuringiensis* with a high degree of diversity, both phenotypic and genotypic among the isolates of *B*. *cereus.*

After the assessment of 38 honey samples from different geographical and floral origins was made, Sinacori et al. [34] reported the presence of 13 species of bacteria where *Bacillus amyloliquefaciens* was the most frequently isolated. Species isolated less frequently were recognized as an environmental contamination. Furthermore, among the microbial species and the botanical/geographical origin of honey no correlation was found. However, the highest microbial diversity was found in multifloral honeys.

Esawy at al. [18] identified six mobile, spore-forming, and Gram-positive facultative aerobic isolates from different honey samples as *Bacillus* spp. also proving a high phenotypic and genotypic variability among *B. subtilis* isolates.

Characterization of microorganisms in Argentinean honeys from different sources performed by Iurlina et al. [35] revealed the presence of *B. cereus* (26%), *B. pumilus* (13%), and *B. laterosporus* (26%) among seventy samples examined.

*Bacillus* spp. were also the most prevalent and constituted more than 67% of bacteria isolated from honey in the study conducted by Wen et al. [36]. Instead of underlining *Bacillus* strains as producers of antibiotics, bacteriocins, or antifungal compounds authors have also indicated *B. anthracis* and many *B. cereus* as toxin-producer strains.

Different members of the *Bacillus* genus (particularly from the *B.* *cereus* group such as *B. thuringiensis*) have been studied by Salazar et al. [37] according to their capability of producing bacteriocins. Bacterial species of the Gram-positive bacteria *L. monocytogenes,* methicillin-resistant *S. aureus,* and the Gram-negative bacteria *P. aeruginosa* were indicated as sensitive to bacteriocins synthesized by *B. thuringensis.* The importance of further investigation into the biological properties of these metabolites because of the wide variety of possible applications was also considered by the authors.

Interestingly one of the identified producing strains was classified as *Paenibacillus* sp. These bacteria are commonly found in soil as rhizobacteria associated with plants roots. However, one specie, namely *Peanibacillus larvae* is the causative agent of diseases lethal to honeybees—American foulbrood. In fact, bee brood is the only established host for *P. larvae* [37,38]. On the basis of the analysis of the sequence of 16S rRNA, we were not able to determine if the producing strain is *P. larvae* or belongs to another species of the genus *Paenibacillus*. In our future studies, we are going to use matrix-assisted laser desorption ionization time-of-flight mass spectrometry (MALDI-TOF-MS) for the final classification of the strain.

Only one sample from present study has demonstrated the growth inhibition of *P. aeruginosa* ATCC 27857 and was described by BLAST as *Staphylococcus* sp., which suggests that this strain is more likely to be an environmental contamination than a natural microflora residing in honey. According to Vázquez-Quiñones et al. [38], inadequate handling (packaging, storage, insects, or even insufficient decontamination) of honey is the main reason for *S. aureus* contamination.

Gene sequence analysis based on 16S rRNA is a commonly used method for bacteria identification and further phylogenetic studies, however, in the case of highly similar sequences as between closely related species, its usefulness is limited. For further differentiation of strains among the members of the *Bacillus* group, more sophisticated methods, such as the aforementioned MALDI-TOF-MS, will be applied in our future research [39].

### Future Research Directions

The possibility of the utilization of microbial compounds isolated from natural sources acting in a bacteriostatic or bactericidal manner seems to be a promising and environmentally acceptable approach. Current findings are promising beginning with the discovery of metabolites active against important human pathogens. Bacteriocins, such as ribosomal synthesized metabolites or bacteriocins-like substances, are especially promising alternatives for antibiotics and could be widely applied in medicine and veterinary settings and for the microbial protection of food products. Firstly, our future research will be the development of simple and reproducible methods of extraction and purification of active metabolites. Chemical structure determination and elucidating the mechanism of action of these agents are also objectives of our future plans. Collection of the active isolates from honey samples will also be maintained—all strains are freely available for other research groups. Furthermore, determination of the spectrum of activity, minimum inhibitory concentration, and cytotoxity tests will be conducted. Finally, growth inhibition of clinical strains of human pathogens, (especially *S. aureus* as activity against these bacteria was the most common among isolated strains) with the most promising metabolites from the collection will be assessed.

## 5. Conclusions

The present study confirmed that Polish honeys of different floral origins are a potent source of bacteria capable of synthesizing substances with an antimicrobial potential. These compounds might be beneficial within different areas such as food biopreservation, medicine (i.e., against antibiotic-susceptible and resistant isolates of *S.* *aureus*), and environmental care. Furthermore, we identified the predominant species residing in honey as belonging to the *Bacillus* group.

Genetic variability among microorganisms isolated from Polish honeys assessed through the study of genomic sequences of 16S rRNA indicates for their environmental background.

## Figures and Tables

**Table 1:** Level of microbial contamination of investigated honeys samples and antimicrobial potential of isolates.

Honey Sample	No. of Colonies per Plate	CFU/ mL of the Product	Activity against *S. aureus* ATCC 25923		Activity against *S. aureus* ATCC 29213		Activity against *S. epidermidis* ATCC 12228		Activity against *E. coli* ATCC 25922		Activity against *P. aeruginosa* ATCC 27857		Activity against *C. albicans* ATCC 10231		Activity against *L. momocytogenes* ATCC 7644	
			No. of Colonies	(%)	No. of Colonies	(%)	No. of Colonies	(%)	No. of Colonies	(%)	No. of Colonies	(%)	No. of Colonies	(%)	No. of Colonies	(%)
3/16	17	34	5	29.41	3	17.65	6	35.29	0	0	0	0	2	11.76	1	5.88
21/16	3136 *	3272	5	25.00	4	20.00	0	0	1	5.00	0	0	3	15.00	0	0
24/16	1520 *	3040	0	0	0	0	0	0	0	0	0	0	0	0	1	5.00
26/16	3	6	1	33.33	1	33.33	1	33.33	0	0	3	100	0	0	1	33.33
28/16	25	50	0	0	0	0	1	4.00	0	0	0	0	0	0	0	0
GBY-GR1	22	44	4	18.18	3	13.65	6	27.27	1	4.55	0	0	2	9.09	7	31.82
J.K/2018	4	8	0	0	0	0	0	0	0	0	0	0	0	0	3	75.00
NGR1	173 *	346	13	65.00	13	65.00	14	70.00	0	0	0	0	0	0	18	10.40
SH2	5	10	2	40.00	2	40.00	1	20.00	0	0	0	0	1	20.00	2	40.00
Spa01	9	18	4	44.44	2	22.22	5	55.56	0	0	0	0	0	0	1	11.11
St01	1	2	1	100	1	100	1	100	0	0	0	0	0	0	0	0
WLK	17	34	3	17.65	4	23.53	6	35.29	0	0	0	0	5	29.41	0	0
**Total**			38		33		41		2		3		13		34	

* 20 randomly selected colonies with a different morphological appearance were selected for further research.

**Table 2:** Growth inhibition of microbial reference strains exhibited by microorganisms isolated from various honey samples **(**“+“ growth inhibition of reference strain was exhibited; “−” growth inhibition of reference strain was not exhibited).

Sample	Honey Source	BLAST	Exhibited Activity						
			*S. aureus* ATCC 25923	*S. aureus* ATCC 29213	S. *epidermidis* ATCC 12228	E. coli ATCC 25922	P. *aeruginosa* ATCC 27857	*C. albicans* ATCC 10231	*L. monocytogenes* ATCC 7644
3_16_1	multiflower	*Bacillus* sp. *(pumilus)*	+	+	+	−	−	+	−
3_16_4	multiflower	*Bacillus* sp. *(pumilus)*	+	−	+	−	−	+	−
3_16_13	multiflower	*Bacillus* sp. *(pumilus/safensis/australimaris)/Microbacterium hydrocarbonoxydans*	+	+	+	−	−	−	−
3_16_15	multiflower	*Bacillus* sp. *(licheniformis)*	−	−	+	−	−	−	+
3_16_17	multiflower	*Bacillus* sp. *(pumilus)*	+	−	+	−	−	−	−
21_16_1	multiflower	*Bacillus* sp. *(pumilus)*	+	+	−	−	−	−	−
21_16_2	multiflower	*Bacillus* sp. *(pumilus/altitudinis/xiamenensis)*	+	+	−	−	−	+	−
21_16_3	multiflower	*Bacillus* sp. *(pumilus/safensis/zhangzhouensis)*	+	+	−	−	−	−	−
21_16_4	multiflower	*Bacillus* sp. *(pumilus/altitudinis/xiamenensis)*	+	−	−	−	−	+	−
21_16_5	multiflower	*Bacillus* sp. *(pumilus)*	+	+	−	−	−	+	−
21_16_6	multiflower	*Peanibacillus* sp.	−	−	−	+	−	−	−
24_16_4	multiflower	*Staphylococcus* sp.	−	−	−	−	−	−	+
26_16_3	multiflower	*Staphylococcus* sp. *(pasteuri)*	−	−	−	−	+	−	−
28_16_5	multiflower	*Microbacterium* sp.	−	−	+	−	−	−	−
GBY_GR1	buckwheat	*Bacillus* sp. *(amyloliqefaciens/valezensis/aryabhattai)*	+	+	+	−	−	−	−
GBY_GR1_2	buckwheat	*Bacillus* sp. *(subtilis)*	+	+	+	−	−	−	−
GBY_GR1_5	buckwheat	*Bacillus* sp. *(aryabhattai/megaterium)*	−	−	+	−	−	−	−
GBY_GR1_11	buckwheat	*Lysinibacillus* sp. *(xylanilyticus)*	−	−	−	−	−	−	+
GBY_GR1_12	buckwheat	*Bacillus* sp. *(licheniformis)*	−	−	−	−	−	−	+
GBY_GR1_13	buckwheat	*Peanibacillus* sp.	−	−	+	+	−	−	+
GBY_GR1_16	buckwheat	*Lysinibacillus* sp. *(fusiformis)*	−	−	−	−	−	−	+
GBY_GR1_19	buckwheat	*Bacillus* sp. *(licheniformis)*	−	−	−	−	−	−	+
GBY_GR1_21	buckwheat	*Bacillus* sp. *(pumilus)*	+	+	+	−	−	+	−
GBY_GR1_22	buckwheat	*Bacillus* sp. *(pumilus/safensis/zhangzhouensis)*	+	−	+	−	−	+	−
JK2_18	multiflower	*Bacillus* sp. *(licheniformis)*	−	−	−	−	−	−	+
JK3_18	multiflower	*Bacillus* sp. *(licheniformis)*	−	−	−	−	−	−	+
NGR1_2	buckwheat	*Bacillus* sp. *(licheniformis)*	−	−	−	−	−	−	+
NGR1_3	buckwheat	*Bacillus* sp. *(kochii)*	−	−	+	−	−	−	+
NGR1_4.1	buckwheat	*Bacillus* sp. *(pumilus)*	+	+	+	−	−	−	+
NGR1_4.5	buckwheat	*Bacillus* sp. *(pumilus/safensis/zhangzhouensis)*	+	+	+	−	−	−	+
NGR1_4.10	buckwheat	*Bacillus* sp. *(pumilus/safensis/zhangzhouensis)*	+	+	+	−	−	−	+
NGR1_7	buckwheat	*Lysinibacillus* sp.	+	−	−	−	−	−	+
NGR1_8	buckwheat	*Bacillus* sp. *(pumilus)*	+	+	+	−	−	−	−
NGR1_9	buckwheat	*Bacillus* sp. *(valezensis/tequilensis)*	−	+	+	−	−	−	−
NGR1_10	buckwheat	*Bacillus* sp. *(licheniformis/aerius)*	−	−	−	−	−	−	+
NGR1_12	buckwheat	*Bacillus* sp. *(licheniformis)*	−	−	−	−	−	−	+
NGR1_13	buckwheat	*Bacillus* sp. *(licheniformis)*	−	−	−	−	−	−	+
NGR1_14	buckwheat	*Peanibacillus* sp./*Bacillus* sp.	−	−	−	−	−	−	+
SH2_1	multiflower	*Bacillus* sp. *(pumilus/altitudinis/xiamenensis)*	+	+	−	−	−	+	−
SH2_3	multiflower	*Bacillus* sp. *(amyloliqefaciens/subtilis/valezensis/aryabhattai)*	+	+	+	−	−	−	−
SH2_4	multiflower	*Bacillus* sp. *(licheniformis)*	−	−	−	−	−	−	+
Spa_01_5	honeydew	*Bacillus* sp. *(pumilus/ altitudinis/xiamenensis)*	+	+	+	−	−	−	−
St 01	multiflower	*Bacillus* sp. *(pumilus/zhangzhouensis)*	+	+	+	−	−	−	−
WLK1_3	multiflower	*Bacillus* sp. *(pumilus/altitudinis/xiamenensis)*	−	+	−	−	−	−	−
WLK1_7	multiflower	*Bacillus* sp. *(pumilus)*	+	+	+	−	−	+	−
WLK1_15	multiflower	*Bacillus* sp. *(pumilus)*	−	−	+	−	−	+	−
